# 1-Benzyl-5-methyl-1*H*-1,2,3-triazole-4-carb­oxy­lic acid

**DOI:** 10.1107/S1600536811056297

**Published:** 2012-01-11

**Authors:** Gai-Gai Wang, Hong Zhao

**Affiliations:** a School of Chemistry and Chemical Engineering, Southeast University, Nanjing 210096, People’s Republic of China

## Abstract

In the title mol­ecule, C_11_H_11_N_3_O_2_, the dihedral angle between the benzene and triazole rings is 76.47 (10)°. The crystal structure exhibits inter­molecular O—H⋯N hydrogen bonds, which lead to the formation of helical chains along [001].

## Related literature

For the synthesis of the title compound, see: El Khadem *et al.* (1968 ▶). For the biological activity of triazole compounds, see: Olesen *et al.* (2003 ▶); Tian *et al.* (2005 ▶). For related structures, see: Xiao *et al.* (2008 ▶); Lin *et al.* (2008 ▶). For structural details of a monohydrate of the title compound, see: Zhao (2009 ▶).
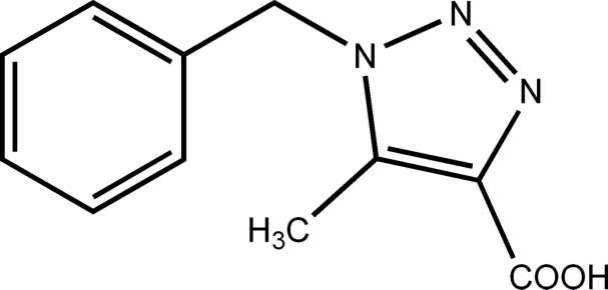


## Experimental

### 

#### Crystal data

C_11_H_11_N_3_O_2_
*M* *_r_* = 217.23
Trigonal, 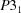

*a* = 10.1178 (7) Å
*c* = 8.9971 (8) Å
*V* = 797.64 (11) Å^3^
*Z* = 3
Mo *K*α radiation
μ = 0.10 mm^−1^
*T* = 293 K
0.20 × 0.18 × 0.15 mm

#### Data collection

Rigaku SCXmini diffractometer
Absorption correction: multi-scan (*CrystalClear*; Rigaku, 2005 ▶) *T*_min_ = 0.921, *T*_max_ = 1.000
8294 measured reflections
2435 independent reflections
1511 reflections with *I* > 2σ(*I*)
*R*_int_ = 0.049

#### Refinement

*R*[*F* ^2^ > 2σ(*F* ^2^)] = 0.053
*wR*(*F* ^2^) = 0.115
*S* = 0.98
2435 reflections
147 parameters
1 restraint
H-atom parameters constrained
Δρ_max_ = 0.13 e Å^−3^
Δρ_min_ = −0.16 e Å^−3^

### 

Data collection: *CrystalClear* (Rigaku, 2005 ▶); cell refinement: *CrystalClear*; data reduction: *CrystalClear*; program(s) used to solve structure: *SHELXS97* (Sheldrick, 2008 ▶); program(s) used to refine structure: *SHELXL97* (Sheldrick, 2008 ▶); molecular graphics: *SHELXTL/PC* (Sheldrick, 2008 ▶); software used to prepare material for publication: *SHELXTL/PC*.

## Supplementary Material

Crystal structure: contains datablock(s) I, global. DOI: 10.1107/S1600536811056297/lr2044sup1.cif

Structure factors: contains datablock(s) I. DOI: 10.1107/S1600536811056297/lr2044Isup2.hkl

Supplementary material file. DOI: 10.1107/S1600536811056297/lr2044Isup3.cml

Additional supplementary materials:  crystallographic information; 3D view; checkCIF report

## Figures and Tables

**Table 1:** Hydrogen-bond geometry (Å, °)

*D*—H⋯*A*	*D*—H	H⋯*A*	*D*⋯*A*	*D*—H⋯*A*
O1—H1⋯N1^i^	0.82	1.91	2.721 (3)	171

Symmetry code: (i) 
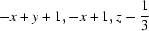
.

**Fig. 1.**
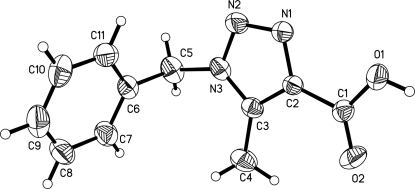
The molecular structure of the title compound. The displacement ellipsoids are drawn at the 30% probability level.

**Fig. 2.**
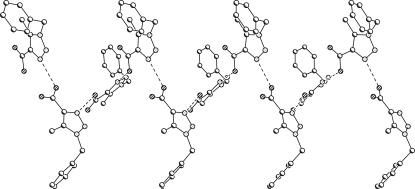
Diagram of the molecules linked into one dimensional chains by O—H···N hydrogen bonds.

## supplementary crystallographic information

### Comment

Triazole-related molecules have attracted considerable attention due to their biological activities (Olesen *et al.*, 2003; Tian *et al.*, 2005). Recently, we have reported a few triazole compounds (Lin *et al.* 2008; Xiao *et al.* 2008). As an extension of our work on the structural characterization of the triazole-related compounds, we report herein the crystal structure of the title compound (Fig. 1), a monohydrate of which has been previously reported (Zhao *et al.*, 2009) .

The dihedral angle between the benzene and tirazole rings is 76.47 (10)°. The crystal structure exhibits intermolecular O—H···N hydrogen bonds which lead to the formation of one-dimensional chains along the [001] direction (Fig. 2.; Table 1).

### Experimental

The title compound was prepared from azidomethylbenzene according to the reported method (El Khadem *et al.* 1968). NiCl_2_ (1 mmol), NaN_3_ (2 mmol) and the title compound (2 mmol) were placed in a thick Pyrex tube (*ca* 20 cm in length). After addition of 2.0 ml of water, the tube was frozen with liquid N_2_, evacuated under vacuum, and sealed with a torch. The tube was heated at 120 °C for 3 days to give colourless prismatic crystals suitable for X-ray analysis.

### Refinement

All H atoms were detected in a difference map, but were placed in calculated positions and refined using a riding motion approxmation, with C—H=0.93–0.97Å and with *U*_iso_(H)=1.2*U*_eq_(C) or 1.5*U*_eq_(C) . The bond distance O—H=0.82 Å and *U*_iso_(H)=1.5*U*_eq_(O).

### Crystal data

**Table:**

C_11_H_11_N_3_O_2_	*D*_x_ = 1.357 Mg m^−^^3^
*M**_r_* = 217.23	Mo *K*α radiation, λ = 0.71073 Å
Trigonal, *P*3_1_	Cell parameters from 1614 reflections
Hall symbol: P 31	θ = 3.2–27.4°
*a* = 10.1178 (7) Å	µ = 0.10 mm^−^^1^
*c* = 8.9971 (8) Å	*T* = 293 K
*V* = 797.64 (11) Å^3^	Prism, colourless
*Z* = 3	0.20 × 0.18 × 0.15 mm
*F*(000) = 342	

### Data collection

**Table:**

Rigaku SCXmini diffractometer	2435 independent reflections
Radiation source: fine-focus sealed tube	1511 reflections with *I* > 2σ(*I*)
graphite	*R*_int_ = 0.049
Detector resolution: 13.6612 pixels mm^-1^	θ_max_ = 27.4°, θ_min_ = 3.3°
CCD_Profile_fitting scans	*h* = −13→12
Absorption correction: multi-scan (*CrystalClear*; Rigaku, 2005)	*k* = −13→13
*T*_min_ = 0.921, *T*_max_ = 1.000	*l* = −11→11
8294 measured reflections	

### Refinement

**Table:**

Refinement on *F*^2^	Primary atom site location: structure-invariant direct methods
Least-squares matrix: full	Secondary atom site location: difference Fourier map
*R*[*F*^2^ > 2σ(*F*^2^)] = 0.053	Hydrogen site location: inferred from neighbouring sites
*wR*(*F*^2^) = 0.115	H-atom parameters constrained
*S* = 0.98	*w* = 1/[σ^2^(*F*_o_^2^) + (0.0487*P*)^2^] where *P* = (*F*_o_^2^ + 2*F*_c_^2^)/3
2435 reflections	(Δ/σ)_max_ < 0.001
147 parameters	Δρ_max_ = 0.13 e Å^−^^3^
1 restraint	Δρ_min_ = −0.16 e Å^−^^3^

### Special details

**Table:**

Geometry. All e.s.d.'s (except the e.s.d. in the dihedral angle between two l.s. planes) are estimated using the full covariance matrix. The cell e.s.d.'s are taken into account individually in the estimation of e.s.d.'s in distances, angles and torsion angles; correlations between e.s.d.'s in cell parameters are only used when they are defined by crystal symmetry. An approximate (isotropic) treatment of cell e.s.d.'s is used for estimating e.s.d.'s involving l.s. planes.
Refinement. Refinement of *F*^2^ against ALL reflections. The weighted *R*-factor *wR* and goodness of fit *S* are based on *F*^2^, conventional *R*-factors *R* are based on *F*, with *F* set to zero for negative *F*^2^. The threshold expression of *F*^2^ > σ(*F*^2^) is used only for calculating *R*-factors(gt) *etc*. and is not relevant to the choice of reflections for refinement. *R*-factors based on *F*^2^ are statistically about twice as large as those based on *F*, and *R*- factors based on ALL data will be even larger.

### Fractional atomic coordinates and isotropic or equivalent isotropic displacement parameters (Å^2^)

**Table:**

	*x*	*y*	*z*	*U*_iso_*/*U*_eq_	
C1	0.5374 (3)	0.4836 (3)	−0.0739 (3)	0.0501 (7)	
C2	0.4072 (3)	0.4056 (3)	0.0273 (3)	0.0440 (6)	
C3	0.2816 (3)	0.4222 (3)	0.0376 (3)	0.0505 (6)	
C4	0.2356 (4)	0.5194 (4)	−0.0452 (4)	0.0751 (9)	
H4A	0.1509	0.4566	−0.1088	0.113*	
H4B	0.3198	0.5920	−0.1042	0.113*	
H4C	0.2064	0.5725	0.0239	0.113*	
C5	0.0466 (3)	0.2884 (4)	0.2051 (3)	0.0709 (9)	
H5A	0.0400	0.3806	0.2120	0.085*	
H5B	0.0333	0.2457	0.3042	0.085*	
C6	−0.0779 (3)	0.1758 (3)	0.1057 (3)	0.0545 (7)	
C7	−0.1626 (4)	0.2174 (4)	0.0197 (4)	0.0715 (9)	
H7	−0.1439	0.3172	0.0231	0.086*	
C8	−0.2762 (4)	0.1118 (5)	−0.0726 (4)	0.0880 (11)	
H8	−0.3333	0.1408	−0.1309	0.106*	
C9	−0.3039 (4)	−0.0348 (4)	−0.0778 (4)	0.0846 (11)	
H9	−0.3792	−0.1056	−0.1405	0.102*	
C10	−0.2215 (4)	−0.0772 (4)	0.0086 (4)	0.0809 (10)	
H10	−0.2417	−0.1775	0.0060	0.097*	
C11	−0.1093 (4)	0.0259 (4)	0.0992 (4)	0.0675 (8)	
H11	−0.0531	−0.0045	0.1573	0.081*	
N1	0.3952 (3)	0.3015 (3)	0.1301 (2)	0.0558 (6)	
N2	0.2667 (3)	0.2531 (3)	0.2037 (2)	0.0650 (7)	
N3	0.1987 (3)	0.3270 (3)	0.1477 (2)	0.0559 (6)	
O1	0.6345 (2)	0.4335 (2)	−0.0625 (2)	0.0648 (6)	
H1	0.7135	0.4916	−0.1068	0.097*	
O2	0.5511 (2)	0.5822 (2)	−0.1573 (2)	0.0758 (7)	

### Atomic displacement parameters (Å^2^)

**Table:**

	*U*^11^	*U*^22^	*U*^33^	*U*^12^	*U*^13^	*U*^23^
C1	0.0500 (16)	0.0416 (15)	0.0522 (16)	0.0180 (13)	−0.0017 (13)	−0.0012 (13)
C2	0.0462 (15)	0.0386 (13)	0.0462 (14)	0.0204 (12)	−0.0054 (12)	−0.0015 (11)
C3	0.0554 (16)	0.0502 (16)	0.0471 (14)	0.0273 (14)	−0.0109 (14)	−0.0081 (13)
C4	0.084 (2)	0.076 (2)	0.085 (2)	0.054 (2)	−0.0121 (19)	0.0013 (18)
C5	0.0624 (19)	0.102 (2)	0.0573 (18)	0.0480 (19)	0.0045 (14)	−0.0167 (17)
C6	0.0455 (16)	0.077 (2)	0.0447 (15)	0.0331 (15)	0.0101 (13)	−0.0023 (14)
C7	0.059 (2)	0.076 (2)	0.088 (2)	0.0399 (19)	−0.0039 (18)	0.0008 (19)
C8	0.060 (2)	0.120 (3)	0.084 (2)	0.044 (2)	−0.0097 (18)	0.013 (2)
C9	0.056 (2)	0.096 (3)	0.078 (2)	0.019 (2)	0.0024 (17)	−0.012 (2)
C10	0.059 (2)	0.069 (2)	0.103 (3)	0.0232 (19)	0.018 (2)	−0.001 (2)
C11	0.061 (2)	0.079 (2)	0.070 (2)	0.0404 (18)	0.0098 (16)	0.0132 (18)
N1	0.0510 (14)	0.0652 (15)	0.0546 (14)	0.0315 (13)	0.0046 (11)	0.0118 (12)
N2	0.0598 (16)	0.087 (2)	0.0519 (14)	0.0393 (15)	0.0036 (12)	0.0153 (14)
N3	0.0499 (14)	0.0745 (16)	0.0479 (13)	0.0345 (13)	−0.0050 (11)	−0.0088 (12)
O1	0.0511 (11)	0.0661 (13)	0.0756 (14)	0.0281 (11)	0.0128 (10)	0.0121 (11)
O2	0.0828 (15)	0.0580 (13)	0.0785 (15)	0.0292 (11)	0.0135 (12)	0.0270 (12)

### Geometric parameters (Å, °)

**Table:**

C1—O2	1.199 (3)	C6—C7	1.368 (4)
C1—O1	1.316 (3)	C6—C11	1.387 (4)
C1—C2	1.465 (4)	C7—C8	1.387 (5)
C2—N1	1.361 (3)	C7—H7	0.9300
C2—C3	1.366 (4)	C8—C9	1.366 (4)
C3—N3	1.343 (3)	C8—H8	0.9300
C3—C4	1.482 (4)	C9—C10	1.357 (5)
C4—H4A	0.9600	C9—H9	0.9300
C4—H4B	0.9600	C10—C11	1.363 (5)
C4—H4C	0.9600	C10—H10	0.9300
C5—N3	1.479 (3)	C11—H11	0.9300
C5—C6	1.500 (4)	N1—N2	1.315 (3)
C5—H5A	0.9700	N2—N3	1.342 (3)
C5—H5B	0.9700	O1—H1	0.8200
			
O2—C1—O1	124.8 (3)	C11—C6—C5	120.1 (3)
O2—C1—C2	122.4 (3)	C6—C7—C8	120.4 (3)
O1—C1—C2	112.8 (2)	C6—C7—H7	119.8
N1—C2—C3	108.7 (2)	C8—C7—H7	119.8
N1—C2—C1	123.2 (2)	C9—C8—C7	119.9 (3)
C3—C2—C1	128.1 (2)	C9—C8—H8	120.0
N3—C3—C2	104.3 (2)	C7—C8—H8	120.0
N3—C3—C4	123.8 (3)	C10—C9—C8	119.9 (4)
C2—C3—C4	131.9 (3)	C10—C9—H9	120.1
C3—C4—H4A	109.5	C8—C9—H9	120.1
C3—C4—H4B	109.5	C9—C10—C11	120.6 (4)
H4A—C4—H4B	109.5	C9—C10—H10	119.7
C3—C4—H4C	109.5	C11—C10—H10	119.7
H4A—C4—H4C	109.5	C10—C11—C6	120.7 (3)
H4B—C4—H4C	109.5	C10—C11—H11	119.7
N3—C5—C6	111.1 (2)	C6—C11—H11	119.7
N3—C5—H5A	109.4	N2—N1—C2	108.6 (2)
C6—C5—H5A	109.4	N1—N2—N3	106.9 (2)
N3—C5—H5B	109.4	N2—N3—C3	111.5 (2)
C6—C5—H5B	109.4	N2—N3—C5	118.5 (2)
H5A—C5—H5B	108.0	C3—N3—C5	129.9 (2)
C7—C6—C11	118.5 (3)	C1—O1—H1	109.5
C7—C6—C5	121.4 (3)		
			
O2—C1—C2—N1	−175.1 (3)	C9—C10—C11—C6	0.5 (5)
O1—C1—C2—N1	5.0 (4)	C7—C6—C11—C10	0.3 (4)
O2—C1—C2—C3	4.5 (4)	C5—C6—C11—C10	−179.6 (3)
O1—C1—C2—C3	−175.4 (3)	C3—C2—N1—N2	0.0 (3)
N1—C2—C3—N3	0.3 (3)	C1—C2—N1—N2	179.7 (2)
C1—C2—C3—N3	−179.4 (2)	C2—N1—N2—N3	−0.2 (3)
N1—C2—C3—C4	179.6 (3)	N1—N2—N3—C3	0.4 (3)
C1—C2—C3—C4	0.0 (5)	N1—N2—N3—C5	176.9 (2)
N3—C5—C6—C7	−109.3 (3)	C2—C3—N3—N2	−0.4 (3)
N3—C5—C6—C11	70.6 (3)	C4—C3—N3—N2	−179.9 (2)
C11—C6—C7—C8	−0.5 (5)	C2—C3—N3—C5	−176.4 (2)
C5—C6—C7—C8	179.3 (3)	C4—C3—N3—C5	4.2 (4)
C6—C7—C8—C9	0.0 (5)	C6—C5—N3—N2	−96.9 (3)
C7—C8—C9—C10	0.8 (5)	C6—C5—N3—C3	78.8 (4)
C8—C9—C10—C11	−1.0 (5)		

### Hydrogen-bond geometry (Å, °)

**Table:**

*D*—H···*A*	*D*—H	H···*A*	*D*···*A*	*D*—H···*A*
O1—H1···N1^i^	0.82	1.91	2.721 (3)	171.

Symmetry codes: (i) −*x*+*y*+1, −*x*+1, *z*−1/3.
